# Diagnostic Utility of Menin Immunohistochemistry in Patients With Multiple Endocrine Neoplasia Type 1 Syndrome

**DOI:** 10.1097/PAS.0000000000002050

**Published:** 2023-05-18

**Authors:** Anna Vera D. Verschuur, Aranxa S.M. Kok, Folkert H.M. Morsink, Wendy W.J. de Leng, Medard F.M van den Broek, Marco J. Koudijs, Johan A. Offerhaus, Gerlof D. Valk, Menno R. Vriens, Bernadette P.M. van Nesselrooij, Wenzel M. Hackeng, Lodewijk A.A. Brosens

**Affiliations:** * Department of Pathology; ‡ Department of Medical Genetics; § Department of Endocrine Surgical Oncology, University Medical Center Utrecht; † Department of Endocrine Oncology, University Medical Center Utrecht Cancer Center, Utrecht University, Utrecht, The Netherlands

**Keywords:** *MEN1*, menin, multiple endocrine neoplasia type 1 syndrome, parathyroid, adenoma, hyperplasia, immunohistochemistry

## Abstract

A clinical diagnosis of multiple endocrine neoplasia type 1 (MEN1) syndrome is usually confirmed with genetic testing in the germline. It is expected that menin protein expression is lost in MEN1-related tumors. Therefore, we investigated the potential of menin immunohistochemistry in parathyroid adenomas as an additional tool in the recognition and genetic diagnosis of MEN1 syndrome. Local pathology archives were searched for parathyroid tumors from patients with MEN1 syndrome and without MEN1, including sporadic, patients with multiple endocrine neoplasia type 2A and hyperparathyroidism-jaw parathyroid tumors. Menin immunohistochemistry was performed and its use to identify MEN1-related tumors was assessed. Twenty-nine parathyroid tumors from 16 patients with MEN1 and 61 patients with parathyroid tumors from 32 non-MEN1 were evaluated. Immunohistochemical nuclear menin loss in one or more tumors was found in 100% of patients with MEN1 and 9% of patients with non-MEN1. In patients with multiple tumors, menin loss in at least one tumor was seen in 100% of 8 patients with MEN1 and 21% of patients with 14 non-MEN1. Using a cutoff of at least 2 tumors showing menin loss per patient, the positive and negative predictive values for the diagnosis MEN1 were both 100%. The practical and additional value of menin immunohistochemistry in clinical genetic MEN1 diagnosis is further illustrated by menin immunohistochemistry in 2 cases with a germline variant of unknown significance in the *MEN1* gene. Menin immunohistochemistry is useful in the recognition of *MEN1* syndrome as well as in the clinical genetic analysis of patients with inconclusive *MEN1* germline testing.

Multiple endocrine neoplasia type 1 (*MEN1*) syndrome is a rare autosomal dominant tumor syndrome caused by a pathogenic germline variant of the tumor suppressor gene *MEN1* on locus 11q.13.^1–3^ This gene encodes the nuclear protein menin, which regulates numerous processes within endocrine organs.^4,5^ Tumorigenesis of *MEN1* syndrome-related tumors follows the Knudson 2-hit model, with inactivation of the wild-type allele by loss of heterozygosity (LOH) or a somatic point mutation.^1,5–7^ Clinically, *MEN1* syndrome translates into a wide variety of endocrine and nonendocrine tumors; however, adenomas in the parathyroid gland, pituitary neuroendocrine tumors, and pancreatic neuroendocrine tumors are the main triad of *MEN1* syndrome-related tumors. A clinical diagnosis is established when at least two of these tumors occur.^1,8^ *MEN1* syndrome is highly suspected if primary hyperparathyroidism develops before the age of 30 years or when a multiglandular parathyroid disease has been found before the age of 40.^1,6,9^ As death is directly related to *MEN1* syndrome-related tumors in 70% of patients with *MEN1* syndrome, early diagnosis by surveillance of patients and their families is pivotal to reduce morbidity and mortality.^9–12^

Proof of a pathogenic germline variant in the *MEN1* gene is considered the gold standard to confirm *MEN1* syndrome. Therefore, a genetic test is offered to patients who fulfill the clinical criteria for *MEN1* syndrome, their families, patients with multigland hyperparathyroidism, or patients with a high suspicion of *MEN1* syndrome.^1^ However, genetic testing is inconclusive in 5% to 25% of patients with a clinical diagnosis and in some patients with high suspicion of *MEN1* syndrome.^1,5^ This is often due to the presence of a variant of unknown significance (VUS) in the *MEN1* gene.^1,9,12,13^ For these patients, the diagnosis and prognosis of *MEN1* syndrome remains uncertain.

*MEN1* mutations often lead to a truncated menin protein resulting in loss of menin expression, which can be visualized by western blotting or immunohistochemistry. Hence, the evaluation of menin expression by immunohistochemistry may be an adjunct to the diagnosis of *MEN1* syndrome patients.^14^ The correlation between loss of menin expression and *MEN1* mutations in the setting of *MEN1* syndrome has been shown in pancreatic tumors.^14,15^ Studies by, Alvelos et al,^16^ Bhuiyan et al,^17^ and Grolmusz et al,^18^ applied menin immunohistochemistry on sporadic and *MEN1* syndrome-related parathyroid tumors. However, these studies were not designed to address the diagnostic potential of menin immunohistochemistry. Hence, consensus on cutoff values, sensitivity, specificity, positive predictive value (PPV), and negative predictive values (NPVs) of menin staining was lacking in these 3 studies.

Hyperparathyroidism is typically the first manifestation of *MEN1* syndrome. To improve the initial recognition of *MEN1* syndrome, we evaluated the diagnostic utility of menin immunohistochemistry in a series of *MEN1* syndrome-related and non-*MEN1* syndrome-related parathyroid tumors. In addition, we describe the application of menin immunohistochemistry in 2 patients with inconclusive *MEN1* germline testing.

## METHODS

### Case Selection and Data Extraction

This study was approved by the Biobank Research Ethics Committee of the University Medical Center Utrecht, The Netherlands. The local pathology archive was searched for resected parathyroid adenomas or hyperplasia (further referred to as tumors) between December 2015 and September 2020 for *MEN1* syndrome-related parathyroid tumors and between April 2017 and September 2020 for non-*MEN1* related tumors. A *MEN1* diagnosis was based on the presence of a (likely) pathogenic germline variant of the *MEN1* gene. The control group included patients with sporadic parathyroid tumors and patients with hyperparathyroidism-associated syndromes such as MEN2A and hyperparathyroidism-jaw tumors. In patients with sporadic tumors, no other clinical symptoms of *MEN1* syndrome were present, preferential DNA analysis was performed, and no variants in the *MEN1* gene were detected. Patients were included in this study if formalin-fixed, paraffin-embedded tumor tissue was available. Finally, parathyroid tumors from patients with a parathyroid tumor and *MEN1* gene VUS were included.

### Immunohistochemistry

4 µm formalin-fixed, paraffin-embedded tissue sections were obtained and heated for 10 minutes at 60°C. Slides were deparaffinized in xylene and rehydrated in 100% ethanol. Endogenous peroxidase activity was blocked with 1.5% H_2_O_2_ in methanol for 15 minutes. For antigen retrieval, the slides were boiled in a pH 6, 10 mM citrate solution for 20 minutes. To block nonspecific binding sites, the sections were incubated for 10 minutes using Pierce Protein-Free Blocking Buffer (Thermo 3757) and were not washed off. Slides were incubated with an antimenin monoclonal antibody (1:400, Abcam ab 92443) diluted in Normal Bright Diluent (UD09-500, ImmunoLogic) for 1 hour at room temperature. The signal was visualized using the BrightVision 2-component detection system Goat AntiMouse/Rabbit IgG HRP (DPVB110HRP, ImmunoLogic), and detected using Bright-DAB (BS04-500, ImmunoLogic). Slides were counterstained with hematoxylin. Archived hematoxylin and eosin-stained slides of all tumor samples were used to assist in the identification of the tumor and scoring of menin immunohistochemistry.

### Immunohistochemistry Scoring

Menin immunochemistry was scored by 3 independent researchers (A.S.K., W.M.H., and L.A.B.) using a fully crossed design. Slides were scored blinded to any clinical information, and the researchers were blinded to each other’s scoring results. Nuclear and cytoplasmic staining of menin were scored separately using stromal cells as an internal positive control. Nuclear menin expression was scored as follows: 0, absent; 1, weak (less intense staining compared with stromal cells); 2, intermediate (equally intense staining compared with stromal cells); 3, strong (more intense staining compared with stromal cells). Cytoplasmic expression was scored as follows: 0 (negative) and 1 (positive). Aberrant menin expression was defined by a nuclear expression score of 0 and 1.

### LOH Analysis

As part of the clinical work-up, LOH analysis was performed in some cases. Therefore, single nucleotide polymorphism (SNP)-array (Infinium CytoSNP-850 K, Illumina) was used according to the manufacturer’s instructions.

### Statistical Analyses

Statistical analyses were performed using *R* version 4.1.3 (R Foundation for Statistical Computing). Differences between patients with *MEN1* syndrome and non-*MEN1* syndrome and tumor characteristics and number of resected tumors per patient were tested using the χ² test or the Mann-Whitney *U* test as appropriate. Sensitivity, specificity, PPV, and NPV of menin immunohistochemistry for detection of *MEN1* syndrome were calculated for different numbers of tumors with menin loss per patient to determine an absolute cutoff value. Interobserver agreement was described in percentages and Fleiss Kappa was used and presented with 95% CI.^19^

## RESULTS

### Patient and Tumor Characteristics

Thirty-one parathyroid tumors were evaluated in 16 patients with *MEN1* syndrome (Table 1). After obtaining tissue sections, 2 cases could not be assessed owing to insufficient tumor tissue, resulting in a total of 29 analyzed tumors. In the non-*MEN1* syndrome group, 61 tumors from patients with sporadic parathyroid tumors (n = 30), MEN2A (n = 1), and hyperparathyroidism-jaw tumor (n = 1) were evaluated (Table 1). In 5 sporadic patients, DNA analysis of peripheral blood did not reveal a variant in the *MEN1* gene. The remaining 25 sporadic patients had a clinical absence of *MEN1* syndrome. The median number of resected and analyzed tumors per patient in the *MEN1* syndrome and non-*MEN1* syndrome groups were 1.5 and 1, respectively (*P* = 1, Supplemental Figure S1A, Supplemental Digital Content 1, http://links.lww.com/PAS/B543 for a boxplot). In 8 patients with *MEN1* syndrome and 14 patients with non-*MEN1* syndrome with multiple tumors (≥2), a median of 3 tumors were resected per patient in both groups (*P* = 0.261) (Supplemental Figure S1B, Supplemental Digital Content 2, http://links.lww.com/PAS/B544 for a boxplot).

**TABLE 1:** Baseline Characteristics Groups With *MEN1* and Non-*MEN1* Syndrome-related Parathyroid Tumors

	*MEN1* syndrome (N = 16); n (%)	Non-*MEN1* syndrome (N = 32); n (%)	*P*
Sex			
Male	9 (56.2)	16 (50.0)	0.920^†^
Age (y)			
Median (Q1-Q3)	34 (29-49)	57 (49-64)	0.002^‡^
Hyperparathyroidism			
Primary	16 (100)	21 (62.3)	0.028^†^
Secondary	0	2 (6.3)	—
Tertiary	0	9 (28.1)	—
Tumors^*^			
Adenoma	4 (25.0)	16 (50)	0.178^†^
Hyperplasia	12 (75.0)	16 (50)	—
Syndrome			
*MEN1*	16 (100)	—	—
MEN2A	—	1 (3.1)	—
HPT-JT	—	1 (3.1)	—
Sporadic, NT	—	25 (78.1)	—
Sporadic, T	—	5 (15.6)	—

* Although parathyroid tumors in *MEN1* syndrome patients are adenomas by definition, this table is based on the initial diagnosis as extracted from the pathology reports.

† χ² test.

‡ Mann-Whitney *U* test.

HPT-JT indicates hyperparathyroidism-jaw tumor syndrome; NT, not tested; T, tested.

### Menin Immunohistochemistry

**FIGURE 1:**
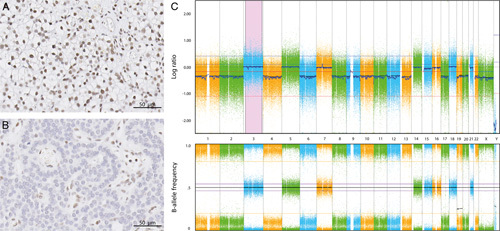
Menin immunohistochemical staining and LOH-analysis. A, Sporadic parathyroid tumor (patient 24) showing preserved nuclear menin expression by immunohistochemistry. B, *MEN1* syndrome-related parathyroid tumor (patient 16) showing absent nuclear menin expression by immunohistochemistry. C, LOH analysis of patient 16, using SNP-array showing *MEN1* LOH.

None of the parathyroid tumors showed positive cytoplasmatic staining. 100% of patients with *MEN1* syndrome and 9.4% patients with non-*MEN1* showed immunohistochemical nuclear menin loss in one or more tumors (Fig. 1A, B, Table 2 and Supplemental Table S1, Supplemental Digital Content 3, http://links.lww.com/PAS/B545; patient characteristics and consensus menin immunohistochemistry scores). In one *MEN1* syndrome tumor with aberrant menin staining (patient 16), LOH analysis showed a loss of chromosome 11q13.1, confirming the genetic loss of *MEN1* (Fig. 1C). Aberrant menin expression in non-*MEN1* syndrome patients was observed in patients with a clinical absence of *MEN1* syndrome, in whom genetic testing was not performed. Using a cutoff of at least one tumor showing menin loss per patient, the PPV and NPV for *MEN1* syndrome diagnosis were 84% (95% CI: 60-97) and 100% (95% CI: 88-100), respectively (Table 3). Among patients with multiple tumors, 100% of patients with *MEN1* syndrome and 21% of patients with non-*MEN1* showed aberrant nuclear menin expression in at least one tumor. The PPV and NPV for the diagnosis of *MEN1* syndrome were 73% (95% CI: 39-94) and 100% (95% CI: 72-100), respectively (Table 3). In case of two tumors per patient with aberrant menin expression, the PPV and NPV were 100% (95% CI: 63-100) and 100% (95% CI: 77-100), respectively (Table 3). These values are similar to the PPV and NPV in case 3 tumors per patient show aberrant menin expression (Table 3).

**TABLE 2:** Patient Characteristics and Consensus Menin Immunohistochemistry Scores of Patients With *MEN1* Syndrome-related Parathyroid Tumors

Patient	Sex	Age	Resected tumors (n)	Excluded tumors (n)	Normal nuclear menin staining (n)	Aberrant nuclear menin staining (n)	Type mutation	Classification
1	F	28	2	1	0	1	Splicing	Likely pathogenic
2	F	38	3	0	0	3	In frame deletion	Pathogenic
3	F	52	3	0	0	3	Frameshift	Pathogenic
4	M	14	1	0	0	1	Nonsense	Pathogenic
5	F	64	4	1	0	3	In frame deletion	Pathogenic
6	M	29	1	0	0	1	In frame deletion	NA
7	M	66	1	0	0	1	Missense	Likely pathogenic
8	M	35	3	0	0	3	Missense	Pathogenic
9	M	31	2	0	0	2	Missense	Pathogenic
10	F	18	1	0	0	1	In frame deletion	Pathogenic
11	F	29	3	0	0	3	Frameshift	Pathogenic
12	M	47	1	0	0	1	Frameshift	Pathogenic
13	F	33	2	0	0	2	In frame deletion	Pathogenic
14	M	16	1	0	0	1	Nonsense	Pathogenic
15	M	48	2	0	0	2	Frameshift	Pathogenic
16	M	54	1	0	0	1	Missense	Pathogenic

NA indicates not applicable.

**TABLE 3:** Sensitivity, Specificity, PPV, and NPV Scores for Patients With *MEN1* and Non-*MEN1* Syndrome-related Parathyroid Tumors

	Aberrant tumors per patient (n)	Sensitivity (95% CI)	Specificity (95% CI)	PPV (95% CI)	NPV (95% CI)
All tumors	1	100 (79-100)	91 (75-98)	84 (60-97)	100 (88-100)
Single tumors	1	100 (63-100)	100 (81-100)	100 (63-100)	100 (81-100)
Multiple tumors	1	100 (63-100)	79 (49-95)	73 (39-94)	100 (72-100)
	2	100 (63-100)	100 (77-100)	100 (63-100)	100 (77-100)
	3	100 (48-100)	100 (66-100)	100 (48-100)	100 (66-100)

As secondary and tertiary hyperparathyroidism have distinct pathogeneses, we repeated the analysis in 16 patients with *MEN1* syndrome and 21 patients with non-*MEN1* syndrome exclusively with primary hyperparathyroidism. PPV and NPV were largely consistent with PPV and NPV considering all types of hyperparathyroidism (Supplemental Table S2, Supplemental Digital Content 4, http://links.lww.com/PAS/B546; for PPV and NPV scores).

### Interobserver Reliability

Kappa was calculated to determine the reliability of the researcher’s scoring per tumor. The Fleiss kappa for parathyroid tumors was 0.818 (95% CI: 0.698-0.937) indicating a near-perfect agreement.

### Menin Immunohistochemistry in Patients With *MEN1* Variants of Unknown Significance

**FIGURE 2:**
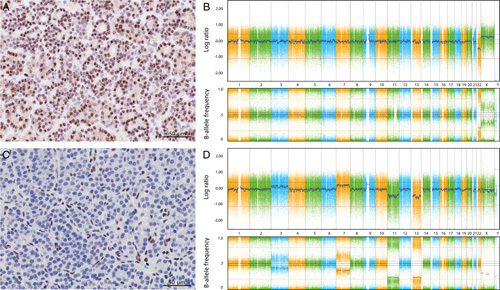
Menin immunohistochemistry and LOH-analysis of 2 parathyroid tumor patients with a clinical suspicion of *MEN1* syndrome and a *MEN1* VUS mutation. One patient (patient 49) showed retained menin expression (A) and LOH analysis using a SNP array showed no copy number variation nor heterozygous loss of *MEN1* (B). The second patient (patient 50) showed aberrant nuclear menin staining by immunohistochemistry (C) and a LOH analysis using an SNP array showed monosomy of chromosome 11, including heterozygous loss of *MEN1* (D).

Finally, parathyroid tumors from 2 patients with clinical suspicion of *MEN1* syndrome and inconclusive genetic results due to *MEN1* gene variants of unknown significance were analyzed by menin immunohistochemistry and for LOH of *MEN1* using SNP-array. One patient with a low clinical suspicion, a VUS was detected and showed preserved menin expression and no LOH (Fig. 2A, B and Supplemental Table S3, Supplemental Digital Content 5, http://links.lww.com/PAS/B547; patient characteristics and consensus menin immunohistochemistry scores). The second patient who had a high clinical suspicion and a VUS, showed immunohistochemical loss of menin as well as LOH (Fig. 2C, D and Supplemental Table S3, Supplemental Digital Content 5, http://links.lww.com/PAS/B547; patient characteristics and consensus menin immunohistochemistry scores).

## DISCUSSION

This is the first study evaluating the use of menin immunohistochemistry as an adjunct in the diagnosis of *MEN1* syndrome. This study shows that the loss of immunohistochemical menin expression in two or more tumors is diagnostic for *MEN1* syndrome in patients with multiple parathyroid tumors. This implies that menin immunohistochemistry can be useful for identifying patients with *MEN1* syndrome in daily pathology practice.

In line with previous results, we found a strong association between the loss of menin expression and the genetic loss of *MEN1*.^15–18^ None of the patients with *MEN1* syndrome showed preserved nuclear menin expression. On inquiry with the antibodies manufacturer, the antibody used in this study binds somewhere between 500 and 615 amino acid of the menin protein. Although we did not test the second hit mutations in the parathyroid tumors with menin loss, the most distal pathogenic *MEN1* germline variant in this cohort of patients with *MEN1* is located at amino acid 522. Probably, the binding region of the menin antibody is found after amino acid 522. Nevertheless, due to the proprietary nature of the antibody immunogens, the exact epitope remains unknown. There are several monoclonal anti-menin antibodies with a more distal located epitope (between amino acid 575 and 615), which we failed to get working on Ventana autostainers in our diagnostic immunohistochemical facility. The polyclonal antibody (Bethyl IHC-00572) also has its epitope between amino acids 575 and 615 and is currently used in our diagnostic immunohistochemical facility. It gives good and comparable results compared with the Abcams ab 92443 antibody.

Unfortunately, in the majority of sporadic cases the absence of *MEN1* syndrome, i.e. a germline *MEN1* mutation, was not excluded. When considering exclusively the patients who had undergone genetic testing, the non-*MEN1* syndrome group was too small to perform a sensitivity analysis. Of the patients with only the clinical absence of *MEN1* syndrome, 3 patients showed aberrant menin expression in one tumor (patients 17, 21, and 39). According to the current guidelines, these patients were not eligible for genetic testing. Aberrant nuclear menin staining in these cases is more likely to be the result of a somatic *MEN1* mutation rather than a germline *MEN1* mutation.

The PPV and NPV presented in this study are consistent with the PPV and NPV in data from Gromlusz’s study on menin immunohistochemistry in (one or more) parathyroid tumors in patients with *MEN1* and non-*MEN1* syndrome.^18^ Genetic testing was performed for all included patients. Based on the extracted data from patients with (n = 14) and without a germline or somatic *MEN1* mutation (n = 30) with at least one tumor with aberrant nuclear menin staining, we calculated the PPV and NPV for *MEN1* syndrome diagnosis to be 80% (95% CI: 56-94) and 100% (95% CI: 87-100), respectively.^18^ However, it should be considered that our institution is a center of expertise for *MEN1* syndrome and is, therefore, a center with a high prevalence of *MEN1*-related tumors. As this information is unknown from the Gromlusz study, the PPV presented in this study might be actually higher than that extracted from the study by Gromlusz and colleagues.

In this study, we analyzed primary, secondary, and tertiary hyperparathyroidism-related tumors. Primary hyperparathyroidism tumors are considered to be due to adenomas, whereas secondary and tertiary hyperparathyroidism-related tumors are considered to begin as hyperplasias. Tumors with biallelic inactivation of *MEN1* resulting in lack of a (functional) menin protein are, by definition, a neoplasia, and are seen in *MEN1* syndrome cases, but also in about 20% of sporadic adenomas.^20^ However, on histologic grounds alone, it is not possible to distinguish between parathyroid adenoma and hyperplastic parathyroid. Although secondary and tertiary hyperparathyroidism-related tumors have different pathogenesis, we analyzed adenomas as well as hyperplastic parathyroids because the loss of menin expression can aid in distinguishing between adenomas and hyperplasia. Notably, 2 of 3 sporadic tumors showing aberrant menin staining were tertiary hyperparathyroidism-related tumors. In these cases, loss of menin expression may be explained by the transition from hyperplasia to adenoma. Moreover, considering the different pathogeneses, we conducted a subanalysis with only primary hyperparathyroidism-related tumors, showing that the PPV and NPVs are in line with our initial results.

In the evaluation of the diagnostic utility of menin immunohistochemistry, we considered several cutoff values. The PPV for the group with multiple tumors was the highest when using a cutoff of two or more tumors with aberrant nuclear staining per patient. As most patients with *MEN1* syndrome present with multiple parathyroid tumors, menin evaluation has the highest value in patients with multiple tumors. The use of menin immunohistochemistry may aid in the clinical management of multiple parathyroid tumors in 3 clinical situations. First, if a *MEN1* VUS is revealed by germline sequencing, our findings indicate that loss of menin expression in at least two parathyroid tumors or the presence of other *MEN1* syndrome pathognomonic tumors (eg, pituitary neuroendocrine tumors or gastropancreatic neuroendocrine tumor) may be an additional argument to consider the VUS as likely pathogenic. In addition, retained menin expression may help to reclassify a VUS as likely benign. Second, as low-middle–income countries may face limited funds and resources for genetic testing, menin immunohistochemistry would be an easy and relatively low-cost alternative to diagnose *MEN1* syndrome. Third, regarding an NPV of 100%, normal menin immunohistochemistry may be a reason to withhold genetic testing in multiple parathyroid tumor patients to reduce health care costs. Furthermore, based on our results, the standard clinical use of menin immunohistochemistry for patients with single parathyroid tumors might be considered as well. As for this group, the PPV was 100% and loss of menin immunohistochemistry could be—in conjunction with other indications—an extra reason to suggest earlier genetic counseling and not wait for a second *MEN1*-related tumor type to occur.

## CONCLUSIONS

Menin immunochemistry can be of added value for diagnosing *MEN1* syndrome. Menin loss, particularly if present in multiple parathyroid tumors, is a strong indicator of an underlying *MEN1* syndrome. In addition, menin immunohistochemistry can serve as a supplementary pathologic tool for patients with inconclusive genetic testing.

## Supplementary Material

## References

[1] Thakker RV Newey PJ Walls GV. Clinical practice guidelines for multiple endocrine neoplasia type 1 (*MEN1*). J Clin Endocrinol Metab. 2012;97:2990–3011. 22723327 10.1210/jc.2012-1230

[2] Chandrasekharappa SC Guru SC Manickam P. Positional cloning of the gene for multiple endocrine neoplasia-type 1. Science. 1997;276:404–407. 9103196 10.1126/science.276.5311.404

[3] Lemmens I Van de Ven WJ Kas K. Identification of the multiple endocrine neoplasia type 1 (*MEN1*) gene. The European Consortium on *MEN1*. Hum Mol Genet. 1997;6:1177–1183. 9215690 10.1093/hmg/6.7.1177

[4] Balogh K Rácz K Patócs A. Menin and its interacting proteins: elucidation of menin function. Trends Endocrinol Metab. 2006;17:357–364. 16997566 10.1016/j.tem.2006.09.004

[5] Lemos MC Thakker RV. Multiple endocrine neoplasia type 1 (*MEN1*): analysis of 1336 mutations reported in the first decade following identification of the gene. Hum Mutat. 2008;29:22–32. 17879353 10.1002/humu.20605

[6] Brandi ML Gagel RF Angeli A. Guidelines for diagnosis and therapy of MEN type 1 and type 2. J Clin Endocrinol Metab. 2001;86:5658–5671. 11739416 10.1210/jcem.86.12.8070

[7] Klein RD Salih S Bessoni J. Clinical testing for multiple endocrine neoplasia type 1 in a DNA diagnostic laboratory. Genet Med. 2005;7:131–138. 15714081 10.1097/01.gim.0000153663.62300.f8

[8] de Laat JM van der Luijt RB Pieterman CR. *MEN1* redefined, a clinical comparison of mutation-positive and mutation-negative patients. BMC Med. 2016;14:182. 27842554 10.1186/s12916-016-0708-1 PMC5109674

[9] de Laat JM van Leeuwaarde RS Valk GD. The importance of an early and accurate *MEN1* diagnosis. Front Endocrinol (Lausanne). 2018;9:533. 30254610 10.3389/fendo.2018.00533 PMC6141626

[10] Al-Salameh A Baudry C Cohen R. Update on multiple endocrine neoplasia type 1 and 2. Presse Med. 2018;47:722–731. 29909163 10.1016/j.lpm.2018.03.005

[11] de Laat JM Tham E Pieterman CR. Predicting the risk of multiple endocrine neoplasia type 1 for patients with commonly occurring endocrine tumors. Eur J Endocrinol. 2012;167:181–187. 22581216 10.1530/EJE-12-0210

[12] van Leeuwaarde RS van Nesselrooij BP Hermus AR. Impact of delay in diagnosis in outcomes in *MEN1*: results from the Dutch *MEN1* Study Group. J Clin Endocrinol Metab. 2016;101:1159–1165. 26751192 10.1210/jc.2015-3766

[13] Kamilaris CDC Stratakis CA. Multiple endocrine neoplasia type 1 (*MEN1*): an update and the significance of early genetic and clinical diagnosis. Front Endocrinol (Lausanne). 2019;10:339. 31263451 10.3389/fendo.2019.00339 PMC6584804

[14] Conemans EB Raicu-Ionita GM Pieterman CRC. Expression of p27(Kip1) and p18(Ink4c) in human multiple endocrine neoplasia type 1-related pancreatic neuroendocrine tumors. J Endocrinol Invest. 2018;41:655–661. 29134609 10.1007/s40618-017-0783-y

[15] Hackeng WM Brosens LA Poruk KE. Aberrant Menin expression is an early event in pancreatic neuroendocrine tumorigenesis. Hum Pathol. 2016;56:93–100. 27342911 10.1016/j.humpath.2016.06.006

[16] Alvelos MI Vinagre J Fonseca E. *MEN1* intragenic deletions may represent the most prevalent somatic event in sporadic primary hyperparathyroidism. Eur J Endocrinol. 2013;168:119–128. 23093699 10.1530/EJE-12-0327

[17] Bhuiyan MM Sato M Murao K. Expression of menin in parathyroid tumors. J Clin Endocrinol Metab. 2000;85:2615–2619. 10902816 10.1210/jcem.85.7.6688

[18] Grolmusz VK Borka K Kovesdi A. *MEN1* mutations and potentially *MEN1*-targeting miRNAs are responsible for menin deficiency in sporadic and *MEN1* syndrome-associated primary hyperparathyroidism. Virchows Arch. 2017;471:401–411. 28597079 10.1007/s00428-017-2158-3

[19] Landis JR Koch GG. The measurement of observer agreement for categorical data. Biometrics. 1977;33:159–174. 843571

[20] Brewer K Costa-Guda J Arnold A. Molecular genetic insights into sporadic primary hyperparathyroidism. Endocr Relat Cancer. 2019;26:R53–R72. 30475215 10.1530/ERC-18-0304

