# Supplementary figures and images for: Diagnostic Utility of Menin Immunohistochemistry in Patients With Multiple Endocrine Neoplasia Type 1 Syndrome
Source: Am J Surg Pathol. 2023 May 18;47(7):785–91. doi: 10.1097/PAS.0000000000002050 (PMC10270278; doi:10.1097/PAS.0000000000002050)

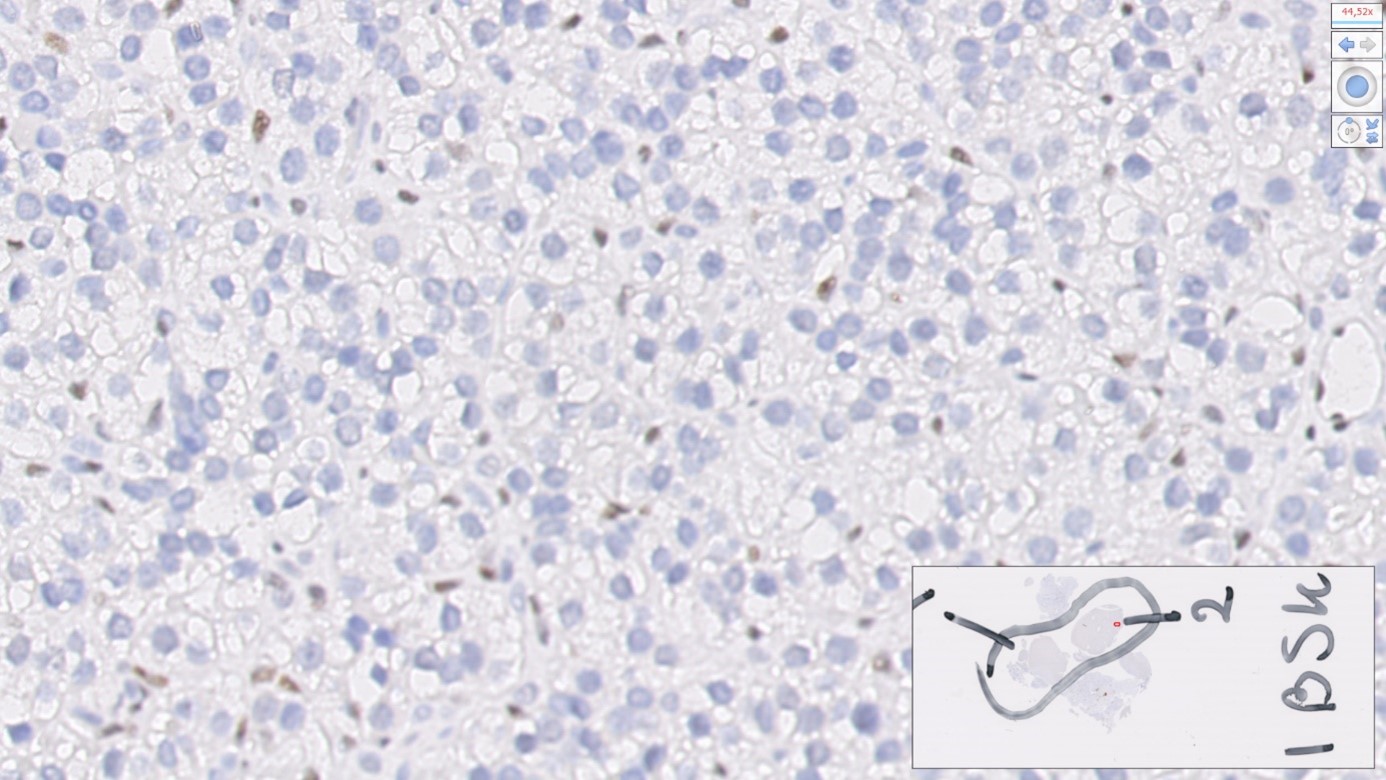

Supplement: Supplementary file 1 [file pas-47-785-s001.jpg]

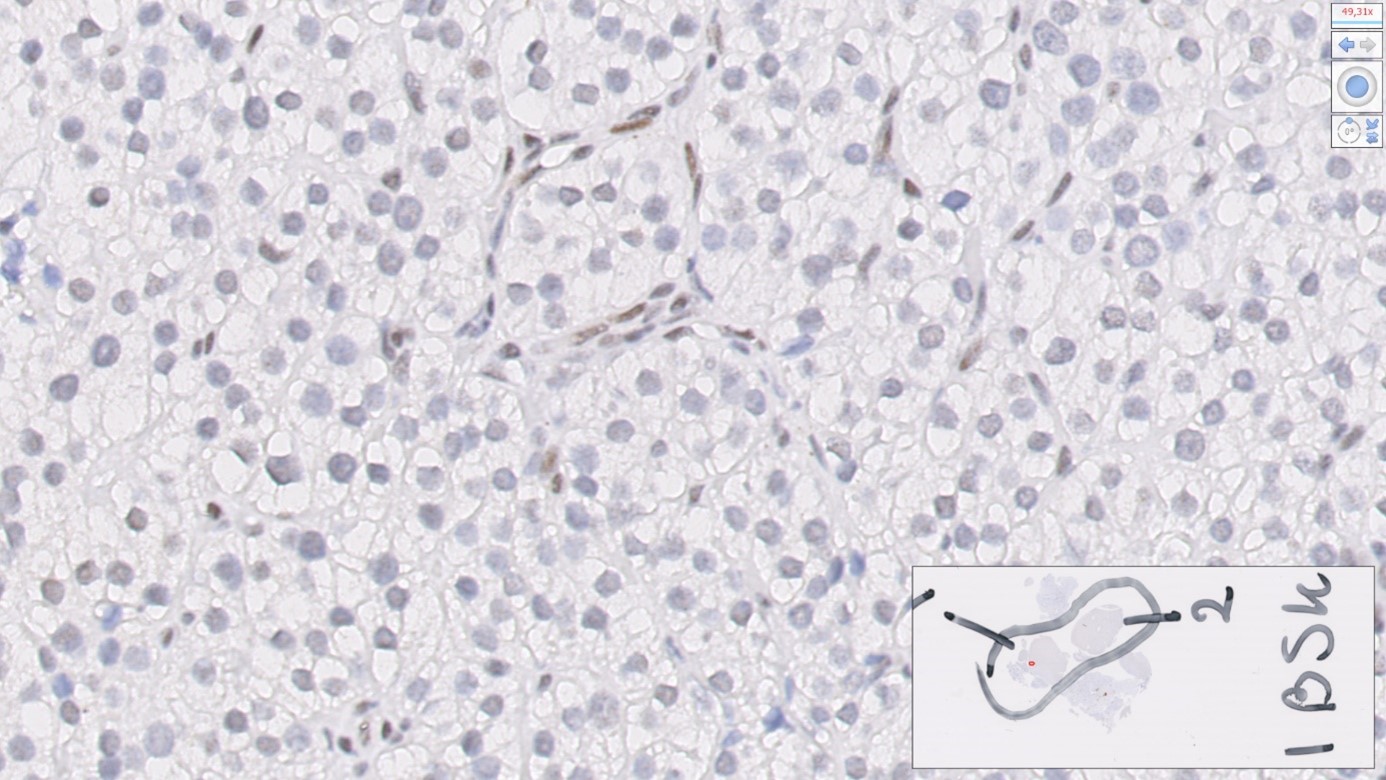

Supplement: Supplementary file 2 [file pas-47-785-s002.jpg]
