# Supplementary material for: Diagnostic Utility of Menin Immunohistochemistry in Patients With Multiple Endocrine Neoplasia Type 1 Syndrome
Source: Am J Surg Pathol. 2023 May 18;47(7):785–91. doi: 10.1097/PAS.0000000000002050 (PMC10270278; doi:10.1097/PAS.0000000000002050)
Supplement: Supplementary file 3 [file pas-47-785-s003.docx]

**Table 1:** Baseline characteristics groups with Multiple Endocrine Neoplasia type 1 (MEN1) and non-MEN1 syndrome related parathyroid tumors.

|  | **MEN1 syndrome (N=16)** | **Non-MEN1 syndrome (N=32)** | **p-value** |
| --- | --- | --- | --- |
| **Sex** |  |  |  |
| Male, n (%) | 9 (56.2%) | 16 (50.0%) | 0.920 ^a^ |
| **Age (years)** |  |  |  |
| Median (Q1-Q3) | 34 (29-49) | 57 (49-64) | 0.002 ^b^ |
| **Hyperparathyroidism** |  |  |  |
| Primary, n (%) | 16 (100%) | 21 (62.3%) | 0.028 ^a^ |
| Secondary, n (%) | 0 (0%) | 2 (6.3%) |  |
| Tertiary, n (%) | 0 (0%) | 9 (28.1%) |  |
| **Tumors*** |  |  |  |
| Adenoma, n (%) | 4 (25.0%) | 16 (50%) | 0.178 ^a^ |
| Hyperplasia, n (%) | 12 (75.0%) | 16 (50%) |  |
| **Syndrome** |  |  |  |
| MEN1, n (%) ♦ | 16 (100%) | - |  |
| MEN2A, n (%) ■ | - | 1 (3.1%) |  |
| HPT-JT, n (%) ● | - | 1 (3.1%) |  |
| Sporadic, NT, n (%)▲ | - | 25 (78.1%) |  |
| Sporadic, T, n (%)✻ | - | 5 (15.6%) |  |

## *Although parathyroid tumors in MEN1 syndrome patients are adenomas by definition, this table is based on the initial diagnosis as extracted from the pathology reports; ♦ MEN1 = Multipele Endocriene Neoplasia Syndroom type 1; ■ MEN2A = Multipele Endocriene Neoplasia Syndroom type 2A; ●HPT-JT = Hyperparathyroidism-jaw tumor syndrome; ▲NT = Not tested, clinical absence of MEN1 syndrome; ✻T = Tested, no *MEN1* mutation found. ^a^ χ² test, ^b^ Mann-Whitney U-test
