# Supplementary material for: Diagnostic Utility of Menin Immunohistochemistry in Patients With Multiple Endocrine Neoplasia Type 1 Syndrome
Source: Am J Surg Pathol. 2023 May 18;47(7):785–91. doi: 10.1097/PAS.0000000000002050 (PMC10270278; doi:10.1097/PAS.0000000000002050)
Supplement: Supplementary file 4 [file pas-47-785-s004.docx]

**Supplemental Digital Content 3: Supplemental Table 2:** Sensitivity, specificity, positive predictive value and negative predictive value scores for patients with Multiple Endocrine Neoplasia type 1 (MEN1) and non-MEN1 syndrome and primary hyperparathyroidism related parathyroid tumors.

|  | **Aberrant tumors per patient (n)** | **Sensitivity (%)** | **Specificity (%)** | **Positive predictive value (%)** | **Negative Predictive value (%)** |
| --- | --- | --- | --- | --- | --- |
| All tumors | 1 | 100 (95% CI: 79-100) | 95 (95% CI: 76-100) | 94 (95% CI: 71-100) | 100 (95% CI: 83-100) |
| Single tumors | 1 | 100 (95% CI: 63- 100) | 100 (95% CI: 81-100) | 100 (95% CI: 63-100) | 100 (95% CI: 81-100) |
| Multiple tumors | 1 | 100 (95% CI: 63-100) | 67 (95% CI: 9-99) | 89 (95% CI: 52-100) | 100 (95% CI: 16-100) |
|  | 2 | 100 (95% CI: 63-100) | 100 (95% CI: 29-100) | 100 (95% CI: 63-100) | 100 (95% CI: 29-100) |
