# Supplementary material for: Diagnostic Utility of Menin Immunohistochemistry in Patients With Multiple Endocrine Neoplasia Type 1 Syndrome
Source: Am J Surg Pathol. 2023 May 18;47(7):785–91. doi: 10.1097/PAS.0000000000002050 (PMC10270278; doi:10.1097/PAS.0000000000002050)
Supplement: Supplementary file 5 [file pas-47-785-s005.docx]

**Table 3:** Sensitivity, specificity, positive predictive value and negative predictive value scores for patients with Multiple Endocrine Neoplasia type 1 (MEN1) and non-MEN1 syndrome related parathyroid tumors.

|  | **Aberrant tumors per patient (n)** | **Sensitivity (%)** | **Specificity (%)** | **Positive predictive value (%)** | **Negative Predictive value (%)** |
| --- | --- | --- | --- | --- | --- |
| All tumors | 1 | 100 (95% CI: 79-100) | 91 (95% CI: 75-98) | 84 (95% CI: 60-97) | 100 (95% CI: 88-100) |
| Single tumors | 1 | 100 (95% CI: 63- 100) | 100 (95% CI: 81-100) | 100 (95% CI: 63-100) | 100 (95% CI: 81-100) |
| Multiple tumors | 1 | 100 (95% CI: 63-100) | 79 (95% CI: 49-95) | 73 (95% CI: 39-94) | 100 (95% CI: 72-100) |
|  | 2 | 100 (95% CI: 63-100) | 100 (95% CI: 77-100) | 100 (95% CI: 63-100) | 100 (95% CI: 77-100) |
|  | 3 | 100 (95% CI: 48-100) | 100 (95% CI: 66-100) | 100 (95% CI: 48-100) | 100 (95% CI: 66-100) |
